# Log2Lose: Development and Lessons Learned From a Mobile Technology Weight Loss Intervention

**DOI:** 10.2196/11972

**Published:** 2019-02-13

**Authors:** Ryan Shaw, Erica Levine, Martin Streicher, Elizabeth Strawbridge, Jennifer Gierisch, Jane Pendergast, Sarah Hale, Shelby Reed, Megan McVay, Denise Simmons, William Yancy, Gary Bennett, Corrine Voils

**Affiliations:** 1 School of Nursing Duke University Durham, NC United States; 2 School of Medicine Duke University Durham, NC United States; 3 Durham Veterans Affairs Medical Center Durham, NC United States; 4 College of Health and Human Performance University of Florida Gainesville, FL United States; 5 Duke Global Health Institute Duke University Durham, NC United States; 6 William S Middleton Memorial Veterans Hospital Madison, WI United States; 7 University of Wisconsin Madison, WI United States

**Keywords:** weight loss, diet, cell phone, mobile phone

## Abstract

**Background:**

Providing financial incentives has gained popularity as a strategy to promote weight loss, but questions remain about how best to utilize them. A promising mobile health strategy provides users with near-real-time financial incentives based on both the process of weight loss (behavioral modification) and actual weight loss. To maximize the impact of this strategy, a methodology is needed to close the gap between the desired behavior and the financial incentive. Leveraging mobile health tools—such as mobile phone apps, cellular body weight scales that transmit data to physicians and researchers, and text messaging for instructions and encouragement—has the potential to close this gap.

**Objective:**

This study aimed to describe the development of an innovative technology-based solution and lessons learned from a feasibility trial—Log2Lose—that encouraged individuals to lose weight by providing near-real-time financial incentives for weight loss and/or dietary self-monitoring.

**Methods:**

We recruited participants (N=96) with a body mass index greater than or equal to 30 kg/m^2^ for a 24-week weight loss trial. Participants received a behavioral intervention of biweekly, in-person group sessions and were instructed to log a minimum number of daily calories in MyFitnessPal and to step on the BodyTrace cellular scale at least twice per week. In a 2×2 design, participants were randomized into 4 groups to receive financial incentives for the following: (group 1) weekly weight loss and dietary self-monitoring, (group 2) dietary self-monitoring only, (group 3) weekly weight loss only, or (group 4) no financial incentives. Diet and weight data from the devices were obtained through application programming interfaces. Each week, we applied algorithms to participants’ data to determine whether they qualified for a monetary incentive (groups 1-3). A text message notified these participants of whether they met weight loss and/or self-monitoring requirements to earn an incentive and the amount they earned or would have earned. The money was uploaded to a debit card.

**Results:**

Our custom-engineered software platform analyzed data from multiple sources, collated and processed the data to send appropriate text messages automatically, and informed study staff of the appropriate incentives. We present lessons learned from the development of the software system and challenges encountered with technology, data transmission, and participants (eg, lost connections or delayed communication).

**Conclusions:**

With consistent and constant validation checks and a robust beta test run, the process of analyzing data and determining eligibility for weekly incentives can be mostly automated. We were able to accomplish this project within an academic health system, which required significant security and privacy safeguards. Our success demonstrates how this methodology of automated feedback loops can provide health interventions via mobile technology.

**Trial Registration:**

ClinicalTrials.gov NCT02691260; https://clinicaltrials.gov/ct2/show/NCT02691260

## Introduction

### Background

More than one-third of the US population has obesity [1]. There are significant physical, psychological, and financial ramifications to this epidemic, including increased risk of type 2 diabetes mellitus, hypertension, and depression and medical cost burden [2]. Weight loss of at least 5% improves functional status, decreases blood pressure, and can reduce the incidence of diabetes by more than 50% over 3 years [3]. Although many weight loss interventions yield an average weight loss of at least 5%, many individuals still struggle to adhere to such interventions, reducing the proportion of individuals who achieve clinically significant weight loss.

Several efficacious interventions have encouraged weight loss by offering incentives. However, a key issue that remains is whether individuals should be offered financial incentives for weight loss, weight loss behaviors, or both. Few studies have examined these questions in the context of weight loss [4]. The few studies that have evaluated incentives for weight loss provided incentives at the end of the study instead of providing incentives for weight loss achieved throughout the program in near real-time when behaviors are being performed [5-9]. This has led to a considerable gap between health behaviors performed and receipt of incentives. Furthermore, previous studies that incented weight loss or dietary self-monitoring did so during in-person sessions that required individuals to turn in self-monitoring records and weigh in [4]. This is logistically feasible but ties the reward to attendance of a specific meeting [10].

### Objectives

On the basis of our hypothesis that rewards need to be proximal to the reward-winning behaviors and that they should not be tied to in-person attendance, our team developed an innovative technology solution—Log2Lose. This mobile health tool encourages individuals to lose weight by providing near-real-time financial rewards for weight loss and/or dietary self-monitoring. This study describes how we approached developing Log2Lose and refined the technology over time based on lessons learned. This information can assist others who are creating similar scalable interventions.

## Methods

### Study Design and Overview

This study was approved by the Duke University Medical Center Institutional Review Board (ClinicalTrials.gov: NCT02691260). To evaluate feasibility of study processes [11], we recruited participants (N=96) through community advertisements with a body mass index greater than or equal to 30 kg/m^2^ for a 24-week weight loss feasibility trial in North Carolina, United States. We sequentially enrolled 3 cohorts (n=34, n=31, and n=31), each consisting of 4 randomized groups: group 1 received incentives for dietary self-monitoring and weight loss, group 2 received incentives for dietary self-monitoring only, group 3 received incentives for interim weight loss, and group 4 received weekly text messages but no incentives. Each group had a mean of 8.75 participants across the 3 cohorts.

Participants received a group-based behavioral intervention focusing on either carbohydrate (cohorts 1 and 3) or calorie and fat (cohort 2) restriction. Each group met with a registered dietitian for 1.5 hours every 2 weeks over 24 weeks. All participants were instructed to record at least 1000 calories (female) or 1200 calories (male) daily via the MyFitnessPal mobile phone app 5 or more days per week, including 1 weekend day, and to weigh themselves at least twice per week on a cellular digital scale (BodyTrace) that transmitted weights to researchers. The rationale for the caloric threshold is as follows. The goal of self-monitoring is to illuminate the discrepancy between goals and actual behavior so that people can make changes to align their goals and behaviors [12]. By tracking at least 1000 or 1200 calories daily, participants will have enough information about their dietary patterns to inform dietary modifications. This threshold is slightly lower than the caloric goal for most people seeking weight loss and allows for small lapses in tracking. The rationale for weighing at least twice per week is that there needs to be enough weight data to calculate weight loss.

Participants in the 3 incentive conditions did not know how much money they could win in any given week. The maximum they could receive over the course of the study was US $300, with a range of US $0 (or $2 in cohort 3) to US $28 weekly. At the end of each week, we sent a text message notifying each participant whether she/he had earned an incentive and, if so, how much. The money was uploaded to a MasterCard debit card via ClinCard, a payment system for clinical trials [6]. Participants in the no-incentive condition also received a weekly text message, which encouraged them to self-weigh and log their dietary intake in MyFitnessPal. During cohorts 2 and 3, we sent an additional text message twice a week to participants with words of encouragement or skill-building tips for weight loss.

### Commercial Software and Hardware Utilized

It is beneficial to utilize commercially available hardware and software to maximize the potential for scaling weight loss programs as well as positively affect adoption of interventions. Using existing solutions allows researchers to capitalize on work already done by designers, front-end software engineers, and hardware manufacturers instead of having to design and develop their own systems. To that end, we utilized products already on the market to deliver the Log2Lose intervention.

**Figure 1:**
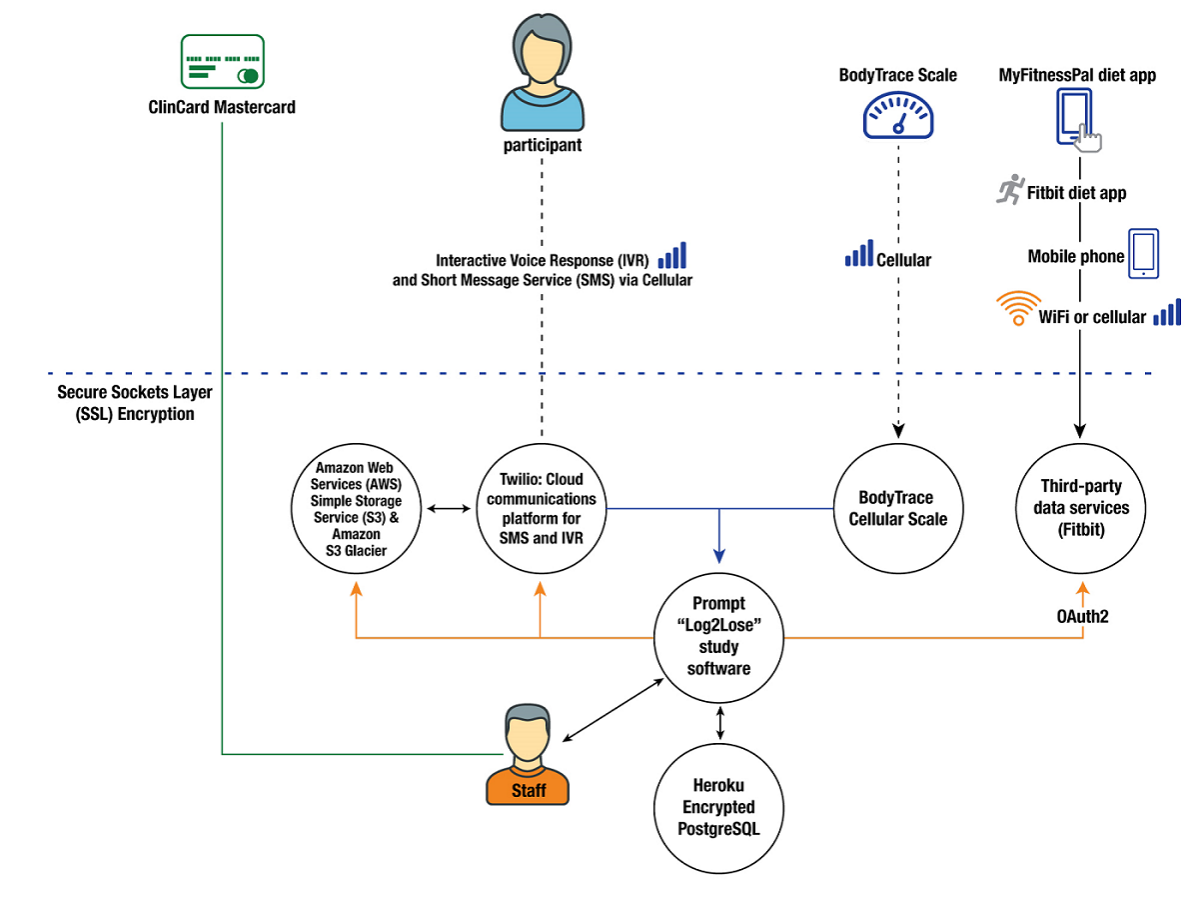
The custom-engineered software platform known as Prompt.

To read and analyze data, we used Prompt (Figure 1) [13], an integrated digital health platform that can test and disseminate evidence-based health solutions and improve participant engagement in clinical and observational studies. Prompt is designed to send and receive data via multiple modalities (text message, interactive voice response, email, or application programming interface [API]). It operates as an interactive, automated tool to help users self-monitor behaviors; receive automated, tailored feedback based on performance; and/or communicate with interventionists. Prompt extends the ability of clinicians and researchers to design and test innovative behavioral interventions and to improve participant engagement in clinical studies. It utilizes tools on Twilio, Heroku, and Amazon S3 and a range of third-party APIs to send, receive, and store data. For this study, Prompt aggregated data via APIs from a dietary app and cellular scale. In addition, it analyzed data per Log2Lose specifications to perform validation checks and to compose Log2Lose text messages.

To retrieve near-real-time diet data, we asked all participants to download the MyFitnessPal and Fitbit dietary tracking apps on their mobile phones. We chose to use the MyFitnessPal app for participant tracking because it provided the appropriate carbohydrate counting interface needed for this study in addition to calorie tracking, whereas Fitbit did not. During the study period, MyFitnessPal did not have an open API (ie, it did not offer an open connection to other software apps). However, MyFitnessPal could share data with the Fitbit app. As a workaround to obtain MyFitnessPal data, we accessed the needed data from the Fitbit API. Study staff helped each participant link his or her MyFitnessPal and Fitbit accounts to each other (linking is a feature on each mobile phone app). Participants added their personal profiles to Prompt, and study staff connected their Prompt profiles to their Fitbit accounts using Open Authorization, a secure authorization protocol.

To retrieve body weight measurements, we provided each participant with a BodyTrace digital scale. These scales transmit readings via cellular service to researchers. We then connected the scale ID to participants’ Prompt profiles. The BodyTrace scale, which connects to a cloud-based database, was chosen for use in this study because (1) it does not require a complicated setup (ie, does not need to be manually connected to a Wi-Fi network); (2) broadband access is not yet ubiquitous across semirural and rural parts of North Carolina, making it possible to use the scale throughout the state; (3) BodyTrace has an open API and provides research staff access to a back-end database, which enables direct collection of data from BodyTrace servers; and (4) it allowed staff to investigate any issues using the BodyTrace back-end database. Although participants were told to weigh themselves at least twice per week (preferably at the beginning and end of each week to maximize weight differences) to permit calculation of weekly weight loss, they were encouraged to weigh daily because individuals who weigh daily tend to lose more weight than those who weigh less frequently [14]. Prompt collected weights daily via the BodyTrace API and time stamped and stored them in a database. Of note, participants could game the system on their home BodyTrace scale by having someone else weigh in for them or by not putting all their weight on the scale. Therefore, our software algorithm detected weights in a single day with a difference greater than 10% and removed those weights to provide some assurance that the measurements came from the same person.

Study staff received access to a multi-factor authentication and password-protected user interface (UI) specifically designed for Log2Lose. This UI allowed staff to view participant information, a list of participants who met the study criteria, a list of participants who earned incentives each week, and the incentive amounts. Although the system automatically analyzed the data and determined who qualified for incentives, because of institutional protocols, study staff had to request money transfers, and a financial administrator had to approve them. This created a delay of approximately 1 day between notification of incentive via text message and receipt of the money on the debit card. Research staff instructed participants on how to use MyFitnessPal and the BodyTrace scale. Participants received tip sheets to help them resolve common error messages that might appear on the digital scale. Research staff were trained to troubleshoot the devices.

### Development of Algorithms to Capture Data

The study design required customized algorithms to collect and analyze data from disparate sources. More than 100 individual algorithms were developed and run automatically, which addressed 2 streams of data (diet app and scale) used differently across 4 randomized groups and differences in payments every week for 24 weeks. Incentives were calculated each week based on the following criteria: (1) whether the participant was sufficiently active based on his or her transmission of data that week; (2) the study week, which determined the incentive amount; (3) the group to which the participant belonged; and (4) whether the participant met the applicable criterion (Table 1).

As 1 validation check, at the end of each week, Prompt reviewed all active participants who were due for an incentive calculation.

**Table 1:** Incentive requirements.

Data source	Requirement for incentive
MyFitnessPal	Log at least 1200 calories for men and 1000 calories for women 5 or more days per week, including 1 weekend day
BodyTrace	Any amount of weight loss, which is calculated as the difference between the first and last weight on the previous 7 days
Prompt	Participant status=active

 For each participant, Prompt read the incentive schedule, the group assignment for that participant, and the diet and/or weight loss results for that week. Prompt then displayed on the UI whether each participant who was due for an incentive qualified for one and, if so, how much. As a second validation check, study staff reviewed the UI at the end of each week to ensure participants qualified or did not qualify for an incentive. They then edited, approved, or did not approve an incentive. Within 24 hours (longer over holidays), the research assistant then scheduled payment of the incentive.

The weekly messages—written and scheduled before the study launched—corresponded with all possible scenarios a participant could face each week. For example, in groups 1-3, participants who met the criteria for an incentive in a particular week received the following message: “Great job on [logging your food and/or losing weight] this week! $[XX] will be added to your debit card. Keep up the good work!,” whereas participants who did not meet the criteria for an incentive in a particular week received the following message: “You did not [log your food or lose enough weight] this week. If you had, you would have earned $[XX]. Please log your food and lose weight for a chance to earn money.” The message to participants who did not meet the criteria emphasized loss to enhance loss aversion [15]. Information in brackets was tailored by week and by the group to which participants belonged. These messages were 160 characters or less so that they appeared in a single message instead of breaking into 2 messages on mobile phones. They were loaded into Prompt and matched to the incentive requirement thresholds. We performed quality assurance testing to validate the message content and timing before the study began.

Participants also received a text message reminder to attend their in-person group session via Prompt. Study staff also sent a message to participants to let them know if there was a cancellation or class rescheduling because of weather. All participants in each condition received the same reminders.

## Results

### Technical Issues Encountered by the Research Team

Log2Lose successfully and consistently automated analysis of incoming data, applied algorithms to those data, and composed and sent text messages on a predetermined schedule. Study staff maintained weekly logs of the technical assistance they provided to participants. From enrollment to completion, technical assistance was needed in screening participants for the appropriate cell phone, aiding with the devices and apps, and running Prompt. Many of the technical issues involved not receiving data from the software apps or devices or being unable to connect to third-party software servers to collect data.

One challenge we encountered was that the cellular scale could only transmit data where a cellular signal existed. Thus, we could not know if data would transmit until after participants enrolled in the study and placed the scales in their homes. Fortunately, this only affected a handful of participants, who were able to move the scale to other locations in their homes for the scales to work.

Another challenge we encountered is that, by the time cohort 3 began, cellular service companies began shutting down 2G networks. A few of our scales were still transmitting on the 2G network, and we had to exchange them for new models. Nevertheless, similar issues could have occurred if we used Bluetooth- or Wi-Fi-enabled scales. As technology evolves, it is a challenge to predict when older technology will be phased out. This unanticipated cost delayed incentives for some participants by a couple of weeks. One lesson learned is that, if technologies are going to be used over a period of time, then they should be bought in batches rather than during study start-up. A couple of participants also stated that they did not like the design of the scale in that they had to turn it on by tapping it before stepping on. This was necessary to tare the scale.

There were also a handful of issues with message formatting. Several times, messages were sent out on different days and at different times than indicated in the protocol because of human error by a member of the research team or a participant. A full description of challenges and solutions is presented in Table 2.

### Patient Acceptability of Technology Solution

Following the intervention, we asked participants to complete a survey regarding their experiences with the technologies. Table 3 describes the questions we asked regarding the diet app and scale. According to self-report, more than half of participants tracked their diet and weight loss over the 6-month study, although participation decreased over time. Participants also reported reading the incentive text messages, and, for the most part, immediately upon receipt.

When comparing follow-up survey responses with the corresponding app data, we found that self-reports generally were consistent. Among the 77 participants for whom follow-up survey responses were available, 18 (18/77, 23%) had no recorded diet app data (Table 3), 14 (14/77, 18%) overestimated the timeframe during which they tracked their diet on the app, and 15 (15/77, 19%) underestimated it. The 46 (46/77, 60%) participants who reported using the BodyTrace scale “every day” averaged 5.4 days per week (SD 1.6) according to the cellular data, the 28 (28/77, 36%) who reported “more than once a week, but not every day” averaged 3.5 days/week (SD 1.5), and the 2 (2/77, 3%) who reported “less than once a week” averaged 1 day a week (SD 1.3). These data suggest high but decreasing adherence to instructions to weigh regularly (at least twice per week) and to log food and drink in MyFitnessPal.

After the study, we interviewed a subset of participants (n=33) from each cohort to ask about their experiences. We purposefully selected individuals who had lost at least 5% of their body weight and those who did not. When asked about their experiences using the cellular scale, most participants stated satisfaction with the scale throughout their use or after a period of adjustment. A few participants said they did not like the scale and did not trust the weight readings because they varied too much day to day. Other individuals reported that the scale was difficult to use at first but that they got used to it. Another complaint was that the scale had to be kept on an even surface. If the scale was placed on an uneven surface, such as carpet, weights were inaccurate. We instructed participants at baseline to place the scale in a place with even surfaces, yet this was still a challenge at times. Furthermore, 1 participant had to exchange a scale 2 times because of unknown issues; these were returned to the manufacturer. Regarding the self-monitoring process, participants mentioned weighing less frequently because they did not enjoy daily weighing or that they became unmotivated when not seeing significant weight loss.

We also asked participants about their experiences using MyFitnessPal. Although 1 individual chose to use paper instead (which would have eliminated them from an incentive for recording food), most participants stated that they liked recording in the app. The most common feature that participants liked was the ability to use the bar scan for commercially available products. However, that was not always possible. If participants went to a restaurant or someone else cooked, they did not always know what ingredients were in the food, making it challenging to record in the app. Complaints from participants who did not use the app were as follows: did not need to use the app because they already knew the calorie content of food, did not like the design of the app, and too many food choices in the app made scrolling difficult.

**Table 2:** Log2Lose software troubleshooting.

Issue type	Example of issue	Potential problem	Solution or solutions implemented
No data	No diet data; no weight data; and incentive text messages do not populate in Prompt	Participant has a previous account and could not remember the password; participant is not tracking or weighing; data are not transmitting from the device or app to server; data are not transmitting from server to Prompt; user accounts are not connected correctly; and programming bug in Prompt	Retrain participant on correct tracking methods; check connection between device/app and third-party server; check API^a^ and Prompt connections; reconnect user accounts; and check and resolve bugs in Prompt
Incorrect information sent to participants	Minor formatting errors in text messages; incorrect incentive amount listed in text message; messages sent out at different days/times than indicated in the protocol; and text messages do not populate in Prompt	Bugs in algorithms and bug in Prompt	Fix/edit original protocols and implement change in Prompt code; manually override information and manually edit and send text messages; and fix bug in Prompt and test fix
Prompt user interface unavailable	Staff unable to log in to user interface	Staff entering incorrect password and bugs in Prompt	Reset passwords and fix bug, test, and implement
Connection error (device/app to the third-party server, to the API, or to Prompt)	Participant tracks in MyFitnessPal, but data are not viewable in Fitbit server, and participant says that she/he weighs, but weight does not show up in server	Prompt reported an error retrieving data from cellular scale and/or Fitbit APIs; participant not tracking; errors appear on the scale; and bugs in Prompt	Check connection between device/app and server; check API-Prompt connections; and resolve bugs in Prompt and test fixes and implement fixes

^a^API: application programming interface.

**Table 3:** Postintervention survey questions.

Question (n=77)	Count, n (%)	Count, n (%)	Count, n (%)	Count, n (%)	Count, n (%)	Count, n (%)
For how long did you track what you ate and drank during this study?	1 month or less, 6 (7%)	Up to 2 months, 9 (11%)	Up to 3 months, 4 (5%)	Up to 4 months, 9 (11%)	Up to 5 months, 6 (7%)	All 6 months, 43 (55%)
I tracked it (diet) with the MyFitnessPal app on my cell phone	Never, 2 (2%)	Rarely, 5 (6%)	Sometimes, 9 (11%)	Often, 11 (14%)	Always, 50 (64%)	—^a^
I tracked it (diet) with a different app on my cell phone	Never, 70 (90%)	Rarely, 1 (1%)	Sometimes, 3 (3%)	Often, 2 (2%)	Always, 1 (1%)	—
How often did you use the BodyTrace scale to weigh yourself during the study?	Every day, 46 (59%)	More than once a week but not every day, 28 (36%)	Less than once a week, 3 (3%)	—	—	—

^a^Indicates there were no answers to select from.

## Discussion

### Principal Findings

Innovative methods are needed to assist individuals in losing weight and maintaining weight loss. As weight loss is a daily undertaking, interventions that reward individuals closer in time to when weight loss behaviors occur are more likely to be effective than those that delay rewards [16]. Incentivizing individuals based on their weight loss behaviors in their daily environments is possible via use of mobile phone technology. This study demonstrates that it is feasible to engage in regular incenting for diet and weight loss in individuals’ daily environments. Although our team incentivized participants with money, many other types of incentives could be transmitted through software systems, including, but not limited to, coupons, discounts, or home delivery of products. In addition, other types of streaming data could be collected from wearable sensors, phone-tethered devices, and sensors placed in the environment.

Our system was sophisticated in its ability to analyze data from multiple sources, to collate and process data to put into appropriate text messages, and to inform study staff of the appropriate incentives. In this development phase of our research, staff spent time monitoring incentives weekly and approving payments. Through our experiences and lessons learned, we anticipate that future versions of the software will be more automated. If the software can apply incentives securely, appropriately, and automatically, then it can reduce the potential for human error as well as costs.

We approached this feasibility study from a developmental standpoint, anticipating frequent corrections to the algorithms and technology platforms. Despite the need for validation checks and test runs, we were able to develop this system in only 4 months. We quickly recognized the importance of having processes in place to help participants resolve bugs and other issues. One of the most important lessons learned is that although we were not able to process incentives immediately because of institutional requirements for processing payments, our system enables this to be achieved in environments with less restrictions. Thus, we have created a methodology that will allow for widespread scaling and dissemination.

### Limitations

First, because this intervention required in-person group meetings, participation was limited to individuals who live close to the medical center. Although research indicates interventions with in-person contact are more efficacious for weight loss [17], requiring continuous in-person contact may affect dissemination potential. Second, because this intervention required a mobile phone with a data plan, access was limited to individuals with a mobile phone and financial means. More than 77% of Americans own mobile phones, and ownership is greater among racial minorities [18]. Third, this study required participants to use diet apps that only work on certain brands of mobile phones, eliminating individuals with less common models of phones (ie, Jitterbug). Fourth, participants may not have recorded dietary intake accurately. As we are focusing on the process rather than the content of recording dietary intake, we believe that incenting this behavior will result in improved outcomes [4]. Finally, because the maximum value of incentives was the same in all 3 incentive conditions, participants in the combined incentive group received half the reward for each singular behavior than the other 2 groups that only rewarded one behavior.

### Conclusions

The study design allowed us to evaluate the feasibility and acceptability of incentivizing dietary self-monitoring on a mobile phone app. These results provide the foundation for a comprehensive, randomized controlled trial to evaluate the impact of incentivizing dietary self-monitoring and weight loss. Due to the proliferation of mobile phones [18] and the increased availability of wireless scales at a more affordable price, this approach looks promising to aid individuals trying to lose weight or maintain weight loss.

## Abbreviations

API: application programming interface
UI: user interface

## Appendix

Multimedia Appendix 1 CONSORT‐EHEALTH checklist (V 1.6.1).
