# Supplementary material for: Log2Lose: Development and Lessons Learned From a Mobile Technology Weight Loss Intervention
Source: JMIR Mhealth Uhealth. 2019 Feb 13;7(2):e11972. doi: 10.2196/11972 (PMC6391641; doi:10.2196/11972)

This image was purchased from istockphoto.com on 12/18/2018 by the Duke University School of Nursing

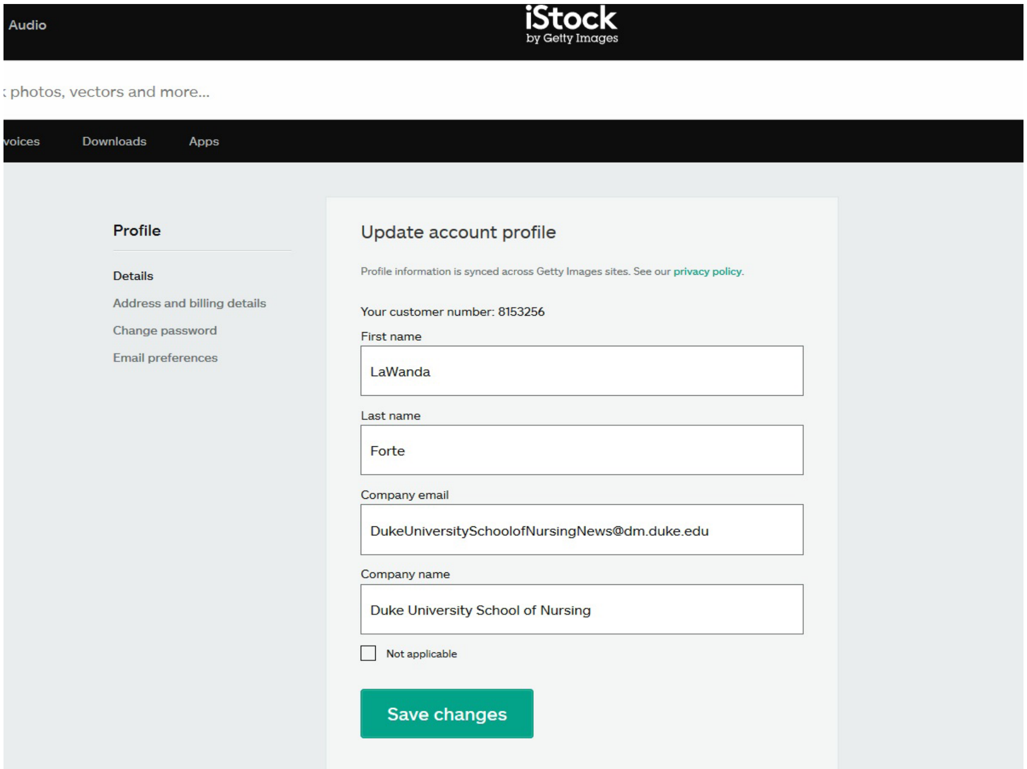

Account #8153256

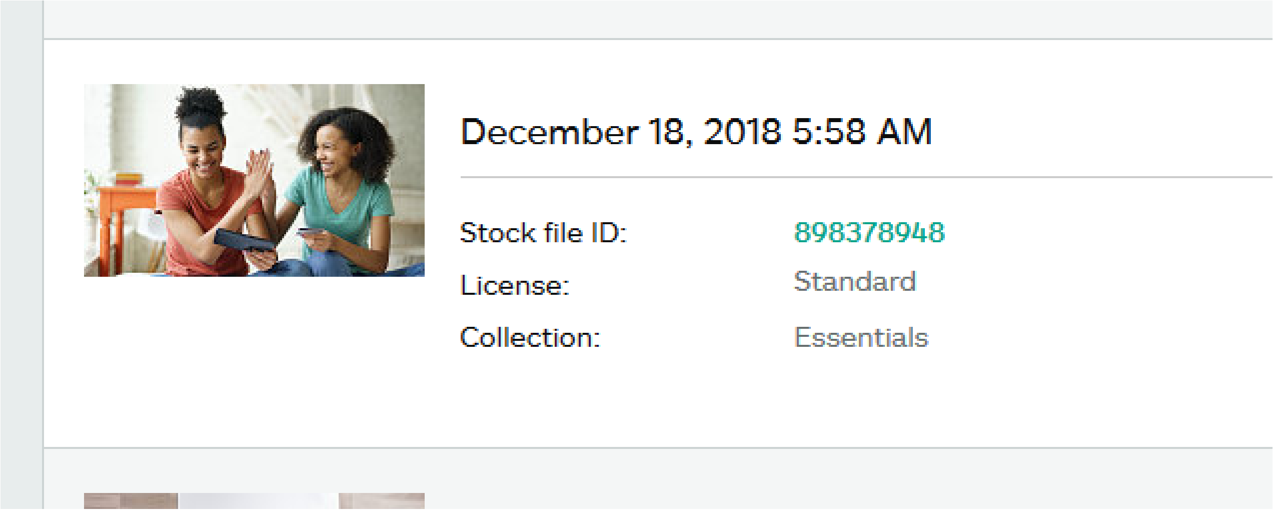

Supplement: Multimedia Appendix 1 [file mhealth_v7i2e11972_fig.pdf]
